# Preparation and Characterization of Dual-Network Multifunctional Hydrogels Based on Peach Gum Polysaccharides: Ultrafast Self-Healing Ability, Favorable Mechanical Tunability, and Controlled Release Properties

**DOI:** 10.3390/gels11040274

**Published:** 2025-04-06

**Authors:** Boyu Liu, Yumeng Han, Zhenqing Zhang, Jianing Hao, Hao Wan, Yongguo Jin, Qi Xu

**Affiliations:** 1 Institute of Advanced Cross-Field Science, College of Life Science, Qingdao University, Qingdao 266800, China; liuby121577@163.com (B.L.); hanym0105@163.com (Y.H.); zhangzhenqing0330@163.com (Z.Z.); 15637597859@163.com (J.H.); hwan@qdu.edu.cn (H.W.); 2 National Research and Development Center for Egg Processing, College of Food Science and Technology, Huazhong Agricultural University, Wuhan 430070, China; jinyongguo@mail.hzau.edu.cn

**Keywords:** oxidized peach gum polysaccharide, dual-network hydrogels, self-healing, controlled release

## Abstract

Natural hydrogels have attracted considerable attention due to advantages of moisturizing, biocompatibility, and plasticity. In this study, a dual-network oxidized peach gum polysaccharide–carboxymethyl chitosan (OPGC) hydrogels with ultrafast self-healing ability was constructed by self-assembly using oxidized peach gum polysaccharide (OPGP) and carboxymethyl chitosan (CMCS). After complete fracture, OPGC hydrogels rapidly self-healed within 30 s due to the dual-network structure formed by the hydrogen bonds between the OPGP molecules and the Schiff base bonds between them and the CMCS. Meanwhile, the hydrogels exhibited good injectability and biocompatibility. With the increase of CMCS from 0.5 wt% to 2.5 wt%, the gel formation time of OPGC hydrogels was drastically shortened from 12 min to 3 min, while the strength and water-holding capacity were enhanced. Furthermore, experimental in vitro and in vivo animal studies demonstrated excellent drug loading capacity of OPGC hydrogels, and the release rate of bactericide could be controlled by adjusting the content of CMCS. The OPGC hydrogels have outstanding properties for potential applications in the health and medical fields.

## 1. Introduction

Hydrogels are porous hydrophilic three-dimensional polymer networks [1]. They are often used in a wide range of applications in the biomedical field, flexible electronic devices, and cell culture [2,3]. Hydrogels could be synthesized by a variety of methods, such as heating, pH adjustment, addition of cross-linking agents, freeze-thawing, and self-assembly [4,5,6]. The self-assembly synthesis method has many advantages, one of which is ease of synthesis. Self-assembled hydrogels can form without introducing cross-linking agents and exhibit excellent biocompatibility [7]. Self-assembled hydrogels are highly porous and uniformly dense to retain more moisture. Partially self-assembled hydrogels have self-healing properties and are capable of spontaneously restoring their structure and function after damage [8]. The self-healing properties of hydrogels can enhance durability and extend their life. Moreover, self-healing hydrogels can improve the adaptability of the material to different working environments. However, improving the self-healing ability of hydrogels has been a challenge, and the rate of self-healing still needs to be improved.

Self-healing hydrogels tend to have multiple network structures consisting of dynamic chemical bonds such as acylhydrazone bonds, imine bonds, hydrogen bonds, and Schiff base bonds [9]. The Schiff base bonds generated by the reaction of the aldehyde groups and the amino groups is simple and inexpensive to synthesize, and it has a high capacity for fracture reconnection [10]. Self-healing hydrogels made of natural materials through Schiff base bonds are safer and show higher repair efficiency [11]. Natural peach crude gum has a high molecular weight and a high degree of branching, is widely available, low- cost, biodegradable, and has good gelation properties. However, its hard texture and poor water solubility limit its application. Peach gum polysaccharides (PGP) can be obtained by hydrolyzing natural crude peach gum. PGP is an acidic polysaccharide mainly composed of arabinose and galactose [12]. PGP also possesses strong antioxidant and antibacterial activities [13,14]. Previous studies have reported that natural PGP has analgesic, lipid-lowering, and immunity-enhancing effects, and can be used as a medicinal herb, food, and industrial raw material [15]. PGP could compose a hydrogel network through hydrogen bonds and hydrophobic interaction. However, the strength of a simple PGP hydrogel network is low. Meanwhile, PGP has a highly branched macromolecular structure that provides abundant functional groups such as hydroxyl and carboxyl groups. The hydroxyl group of PGP could be oxidized to aldehyde groups, thus expanding the gel-forming potential. Natural chitosan, as an alkaline polysaccharide in nature, is mostly obtained from crustaceans in nature [16]. Carboxymethyl chitosan (CMCS) is a chitosan derivative that contains a large number of carboxyl groups [17]. Various medical benefits are provided by it, such as promotion of wound healing and hemostasis, inhibition of scarring, and analgesic effects [18]. It is ideally suited for making medical wound dressings. It is non-toxic, safe, comes from a wide range of sources, and can be used in a large number of applications in the manufacture and improvement of biological products [19]. At the same time, it is also a very good hydrogel material.

In this study, PGP was oxidized to form oxidized peach gum polysaccharide (OPGP), which established the dual-network structure formed by the hydrogen bonds between the OPGP molecules and the Schiff base bonds between them and the CMCS. The gelation time, morphology, physical properties, injectability, and self-healing ability of the hydrogels were investigated. Amoxicillin (AM) and silver ions (Ag^+^) were encapsulated in the hydrogels, and the drug-carrying capacity, drug-release capacity, and antimicrobial effect were characterized in vitro and in vivo in animals. The results showed that the oxidized peach gum polysaccharide–carboxymethyl chitosan (OPGC) hydrogel was injectable and had fast self-healing ability. Its potential as a carrier and medical dressing was further validated by drug-carrying and drug-release experiments.

## 2. Results and Discussion

### 2.1. Synthesis and Characterization of OPGC Hydrogels

PGP was oxidized by NaIO_4_ to increase the aldehyde group content of OPGP. The effect of adding different ratios of oxidizers on the aldehyde group content of OPGP was also investigated. As shown in Table 1, unoxidized PGP has a low aldehyde group content and cannot be measured accurately. OPGP aldehyde group content was greatly increased by NaIO_4_ oxidation treatment. The increased content of OPGP aldehyde groups was beneficial for the subsequent synthesis of OPGC hydrogels. At the same oxidation time and oxidation temperature, the content was different for the aldehyde groups of OPGP when the ratio of NaIO_4_ to PGP was different. When the ratio of NaIO_4_ to PGP was 0.5:1, the OPGP aldehyde group content was 0.78 mmol/g. Changing the NaIO_4_ to PGP ratio from 0.5:1 to 2.5:1 significantly increased the aldehyde group content to 3.30 mmoL/g with increasing oxidant (*p* < 0.05). When the oxidizer addition ratio was increased to 3:1, the aldehyde group content decreased to 3.10 mmol/g, indicating that the oxidant addition ratio was disproportionate, which was not conducive to the increase of aldehyde group content in OPGP. Therefore, NaIO_4_ with a PGP addition ratio of 2.5:1 was used to prepare OPGP in the subsequent oxidation experiments.

The OPGP obtained by oxidation treatment still had the ability to form its own hydrogel network alone. However, its gelation properties were weak, the gel formation time was excessive, and the gels had a certain degree of mobility (Figure S1). It could not maintain a stable gel morphology. Here, CMCS was added to OPGP to improve its gel properties.

**Figure 1:**
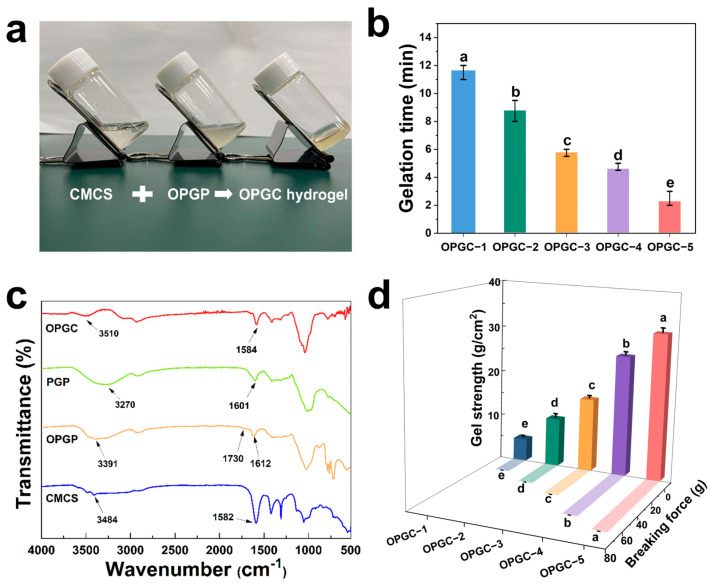
(**a**) Construction of oxidized peach gum polysaccharide–carboxymethyl chitosan (OPGC) hydrogels; (**b**) Gel formation time of OPGC-1/OPGC-2/OPGC-3/OPGC-4/OPGC-5; (**c**) Infrared profiles of oxidized peach gum polysaccharides, carboxymethyl chitosan, and OPGC; (**d**) Gel strength and breaking force of OPGC-1/OPGC-2/OPGC-3/OPGC-4/OPGC-5. The OPGC hydrogels were named OPGC-1/OPGC-2/OPGC-3/OPGC-4/OPGC-5 according to the carboxymethyl chitosan (CMCS) concentration (0.5/1/1.5/2/2.5 wt%). Different letters represent significant differences (*p* < 0.05).

The process of preparing OPGC hydrogels using one-step synthesis was shown in Figure 1a. The OPGP gels were fluid and viscous, whereas the individual CMCS solution exists as a liquid. The CMCS solution was added to the OPGP gels, and the OPGC hydrogels were rapidly formed by rapid stirring at 25 °C. The ideal gelation time is critical for hydrogel application [20], so the gelation time of OPGC hydrogels with different amounts of CMCS addition was investigated. The OPGC gelation time was shortened from 12 min to 3 min with an increase of the CMCS content from 0.5 wt% to 2.5 wt% (Figure 1b). By adjusting the content of CMCS, gel formation was accelerated [21]. When the CMCS content was lower than 0.5 wt%, it failed provide enough carboxyl groups for each hydrogel to form sufficient physical or chemical bonds to maintain a stable shape. OPGC hydrogels can be molded in a desired time frame, which thereby provides a broad space for the practical application of OPGC hydrogels.

The structures of the organic compounds and hydrogels were analyzed using FTIR spectroscopy. The FTIR spectra of CMCS, PGP, OPGP, and OPGC hydrogels are shown in Figure 1c. The peaks in the range of 3200–3650 cm^−1^ correspond to the stretching vibration of O-H. For CMCS, the peak at 1582 cm^−1^ was attributed to the N-H bending of the amino groups. PGP and OPGP peaks near 1600 cm^−1^ were characteristic peaks of the -COO stretching vibration [22]. Compared to PGP, the weakening of the O-H peak in OPGP indicates hydroxyl consumption, and a new absorption peak appeared at 1730 cm^−1^ corresponding to the stretching vibration of C=O of the aldehyde groups [23]. A shift in the O-H peak of OPGP from 3391 cm^−1^ to 3510 cm^−1^ was observed in the OPGC hydrogels, which suggested the formation of intermolecular hydrogen bonds [24]. Meanwhile, the newly appeared C=N absorption peak at 1650 cm^−1^ was the characteristic peak of a Schiff base bond [25]. The Schiff base bonds formed between the aldehyde groups contained in OPGP and the amino groups contained in CMCS were reversible covalent bonds. Presumably the breakage and reconnection of Schiff base bonds provide the hydrogels with the self-healing ability of dynamic reconfiguration, and together with the hydrogen bonds among the OPGP molecules, form the dual-network structure of OPGC hydrogels.

### 2.2. Mechanical Properties of OPGC Hydrogels

The maximum tensile force that can be withstood by the external force is indicated as the breaking force; both it and the gel strength are important indicators of the hydrogels’ mechanical properties. The mechanical properties of OPGC hydrogels were investigated using a texture analyzer. As shown in Figure 1d, the breaking force and gel strength of the hydrogels exhibited a concentration-dependent effect on the CMCS content. The breaking force and gel strength of the hydrogels progressively enhanced with an increase in CMCS addition from 0.5 wt% to 2.5 wt%. With the increase of CMCS content, the fracture force gradually increased, which elongated the tensile deformation of the hydrogels and imparted greater toughness to the hydrogels. This increased toughness makes the OPGC hydrogels less likely to break. This could be attributed to the fact that increasing the addition of CMCS provides more amine and hydroxyl groups. At the same time, oxidized peach gum contains high amount of aldehyde groups, thus forming more Schiff bases with the amine of CMCS.

These chemical bonds enhanced the gel network structure and improved the gel strength and breaking force. It is noteworthy that the increase in CMCS addition was not only associated with an improvement in the strength and toughness of the hydrogels but also with an acceleration in the gel formation. More CMCS content resulted in a shorter gel formation time and a higher gel strength in OPGC hydrogels. High-strength hydrogels can be obtained in a short period of time. Therefore, the problem of the inability of most hydrogels to balance gel strength and gel formation speed is solved. OPGC hydrogels are more advantageous for practical applications [26]. Hydrogels with ideal mechanical properties can be quickly produced for different application scenarios.

**Figure 2:**
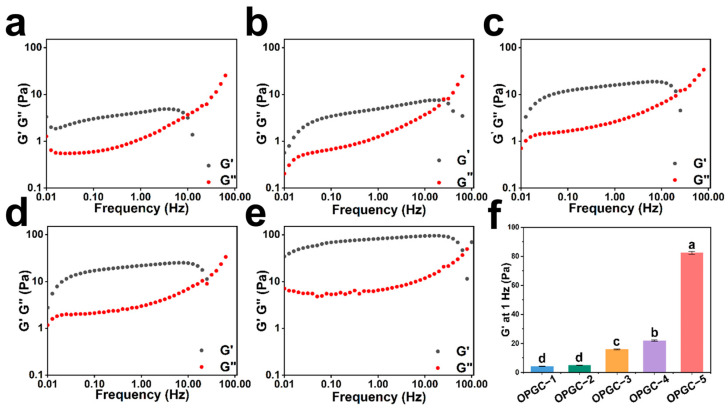
(**a**–**e**) Storage modulus (G’) and loss modulus (G’’) of OPGC-1/OPGC-2/OPGC-3/OPGC-4/OPGC-5. (**f**) Storage modulus (G’) of OPGC-1/OPGC-2/OPGC-3/OPGC-4/OPGC-5 at a frequency of 1 Hz. Different letters represent significant differences (*p* < 0.05).

The phase transition of the gel was characterized by rheological analysis, and the formation of a gel was determined based on a comparison of the values of the storage modulus (G’) and the loss modulus (G”). By adjusting the amount of CMCS, a series of OPGC hydrogels with different mechanical properties can be synthesized. Dynamic frequency scanning rheometry was used to investigate the mechanical properties of OPGC hydrogels. As depicted in Figure 2a–e, OPGC hydrogels exhibited frequency-dependent viscoelastic behavior. The G’ was noticeably exceeded by the G” over the entire frequency range, even with 0.5 wt% CMCS in OPGP, which confirms the formation of hydrogels. For the different hydrogels, with the increase of CMCS concentration from 0.5 wt% to 2.5 wt%, both the G’ and G” were observed to increase markedly. It is inferred that the network of OPGC hydrogels became denser. As depicted in Figure 2f, the order of G’ at 1 Hz frequency was 5G’ > 4G’ > 3G’ > 2G’ > 1G’, meaning the law of change was consistent with the texture results. It was further shown that the OPGC hydrogel strength gradually increased. Further, the crossover frequency of the phase transition points was utilized to evaluate the hydrogels strength, with the crossover point at G’ equal to G” indicating that the hydrogel had undergone a phase transition. The addition of CMCS enhanced the phase transition frequency, and a gel strength of 10 Hz was achieved with the addition of 0.5 wt% CMCS. With the CMCS addition being gradually enhanced to 1, 1.5, 2, and 2.5 wt%, the phase transition frequency correspondingly increased to 19.95, 25.12, 31.62, and 63.10 Hz, which implied that the gel strength enhanced with the increase in CMCS addition. The enhancements in the phase transition point of OPGC hydrogels confirmed the breaking force variation rule, and the CMCS content was closely related to the hydrogels’ strength. Increasing the volume of CMCS added caused the OPGC hydrogels’ dual-network structure to become denser and stable.

### 2.3. Hydration Properties of OPGC Hydrogels

**Figure 3:**
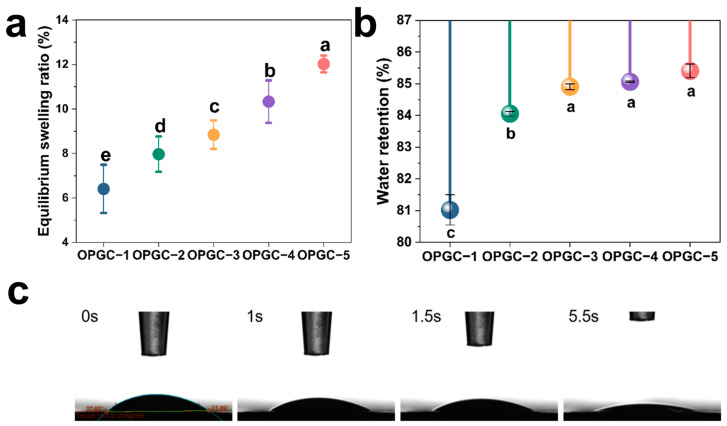
(**a**) The swelling rate of OPGC-1/OPGC-2/OPGC-3/OPGC-4/OPGC-5; (**b**) Water retention rate of hydrogels dried at 37 °C for 24h; (**c**) Optical water contact angle of OPGC hydrogels. Different letters represent significant differences (*p* < 0.05).

Hydrogels usually have excellent water holding and swelling properties. PBS buffer (0.01 mol/L, pH 7.4) at 37 °C was used to immerse OPGC hydrogels for the investigation of equilibrium swelling properties. The swelling equilibrium was reached by all hydrogels at 12 h immersion, proving that the OPGC hydrogels had good equilibrium swelling properties and strong water absorption capacity. As shown in Figure 3a, the hydrogels’ swelling rate gradually increased from 6.4% to 12.3% with the inclusion of more CMCS in the OPGP, resulting in a nearly doubled swelling rate value. The increase in CMCS content resulted in more amino groups, which formed more Schiff bases with aldehyde groups in OPGP. The OPGC hydrogels formed a denser dual-network structure, denser pores, and increased specific surface area and micro space, which endowed the hydrogels with a faster and stronger water absorption ability. Crude peach gum has low water content and low water retention capacity, while the hydrogel composed of OPGP had a strong water retention capacity.

To investigate the water retention capacity of the hydrogels, a series of OPGC hydrogels were subjected to study, as depicted in Figure 3b. All OPGC hydrogels retained more than 80% of their weight. This shows that the OPGC hydrogels were highly capable of retaining water. Moreover, as the CMCS concentration increased, the mass of the hydrogels retained correspondingly increased, implying that the hydrogels had an enhanced water retention capacity. The denser internal network of the hydrogels was also indicated, enabling it to lock in more water, which corresponds to the results of the swelling rate test. This feature endowed the OPGC hydrogels with a superior water retention ability to effectively lock in moisture and offered it superior moisturizing performance. As a result, the excellent swelling rate and water retention capacity of the OPGC hydrogels meant they could absorb more water in a shorter period of time and retain water in the environment for a long period of time.

As depicted in Figure 3c, the water contact angle was used to examine the hydrophilicity properties of the OPGC hydrogels. A hydrogel surface was hydrophilic when the contact angle between a water droplet and a hydrogel was less than 90°. Conversely, when the contact angle between a water droplet and a hydrogel was more than 90°, it was hydrophobic [27]. The initial contact angle between the water droplets and the OPGC hydrogels was measured at 37.97°, indicating that the OPGC hydrogels had good hydrophilicity. Subsequently, the contact angles of samples were observed to decrease, and the droplets were gradually absorbed and almost penetrated into the gel within 5.5 s, indicating that the hydrogels had the capacity to quickly absorb the droplets [28]. Further confirmation was provided that the hydrogels possessed superior water-absorbing capacity. The OPGC hydrogels were endowed with excellent water absorption and water retention capacity by the formation of a dense three-dimensional network through Schiff base bonds and hydrogen bonds.

### 2.4. Morphology Analysis of OPGC Hydrogels

**Figure 4:**
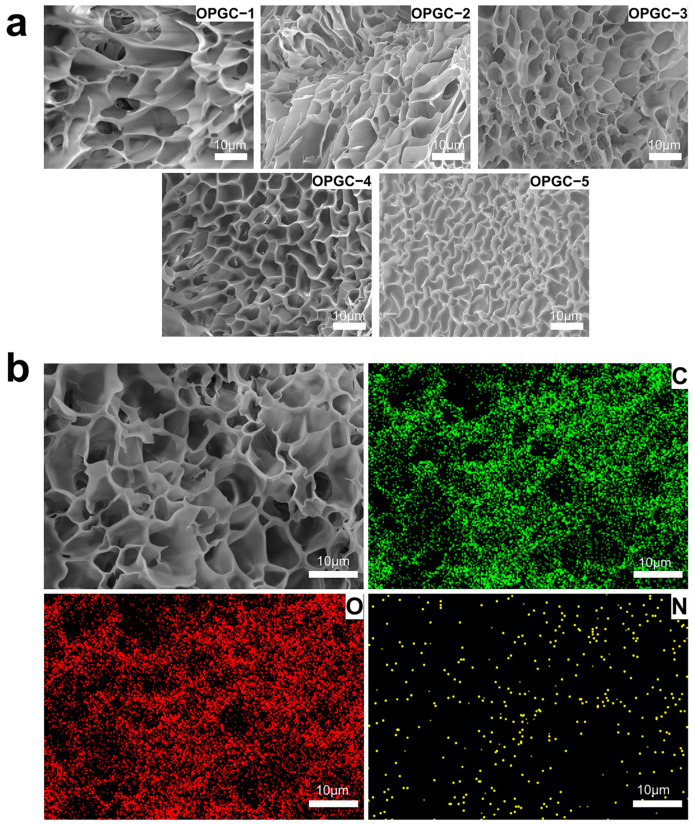
(**a**) SEM images of OPGC-1/OPGC-2/OPGC-3/OPGC-4/OPGC-5; (**b**) Hydrogels EDS plot at 1.5 wt% carboxymethyl chitosan addition; scale bar: 10 μm.

The micro-morphology of the hydrogels was observed by scanning electron microscopy (SEM). As shown in Figure 4a, an obvious microscopic network structure and porous structure were observed in the OPGC hydrogels. With the increase of CMCS addition, the gel pores gradually narrowed, and a denser network structure was formed by the OPGC hydrogels, which implies better gel properties. It confirms the results of the previous analysis. As the concentration of CMCS increased, more amino groups were provided, leading to the formation of more Schiff base bonds with the aldehyde groups on the OPGP, which was responsible for the denser dual-network structure. Therefore, with the increase of CMCS addition in the OPGP, the OPGC hydrogels had stronger water holding capacity, gel strength, and breaking force. As shown in Figure 4b, the element mapping demonstrates that the C, O, and N elements were uniformly distributed throughout the OPGC hydrogels, which constitutes the stable structural basis of hydrogels and gives hydrogels uniform mechanical properties. Meanwhile, the distribution overlapped with the pore distribution observed in the SEM images. The formation of homogeneous cross-links between OPGP and CMCS, which led to the creation of a homogeneous and stable dual-network structure, was further confirmed by the two types of images. An increase in gel strength resulted from this structure, which was found to be consistent with the results of gel strength, breaking force, and rheological analysis.

### 2.5. Self-Healing Ability and Injectability of OPGC Hydrogels

**Figure 5:**
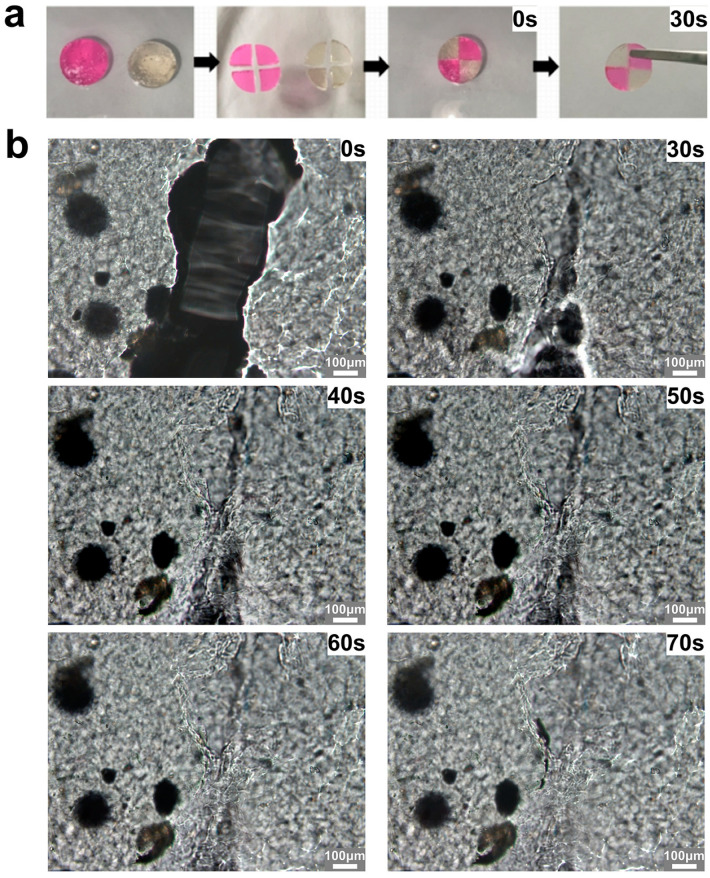
(**a**) Macroscopic self-healing pictures of OPGC hydrogels, The pink hydrogel is an OPGC hydrogel stained with rhodamine B.; (**b**) OPGC hydrogels’ microscopic self-healing process.

Mechanical damage is prone to occur in hydrogels, resulting in breakage, which causes great inconvenience for practical applications. As depicted in Figure 5a, excellent self-healing ability was endowed upon OPGC hydrogels by the Schiff base bonds’ dynamic cross-linking. After hydrogel staining, each hydrogel was cut into four equal parts and reassembled. The newly assembled hydrogels could be restored to their whole form after 30 s. Previously reported self-healing gels tend to require a long recovery time, usually up to several hours. For example, hydrogels crosslinked by dynamic cross-linking agents, which are based on non-covalent interactions, had to be heat-treated at 70 °C to achieve reconstruction of the gel network within 3.5 h [29]. Hyaluronic acid-based hydrogels, which have the advantage of ensuring reliable self-healing at low temperatures (−20 °C), require 5 min at room temperature to complete self-healing [30]. A polymer-based photocrosslinked hydrogel was able to heal by light-induced repair within 60 s, which is closest to the healing speed of OPGC hydrogels [31]. Notably, OPGC hydrogels could achieve ultra-fast self-healing within 30s at room temperature, based on natural macromolecules and independent of any cross-linking agents or external conditions. The results show that OPGC hydrogels possess strong self-healing ability.

As depicted in Figure 5b, rapid healing of hydrogel fractures was further observed under the microscope. The re-crosslinking of the hydrogel gaps occurred at 30 s, and it was noted that the extent of the fracture was further reduced within 60 s. The crack completely reintegrated, and an almost complete three-dimensional gel network reformed at 70 s. The OPGC hydrogels’ self-healing ability derived from the breakage and rapid reconnection of Schiff base bonds and hydrogen bonds upon mechanical damage, enabling the hydrogel breaks to rapidly heal and return to full form [32]. The self-healing ability of OPGC hydrogels is mainly attributed to the dynamic remodeling of Schiff base bonds [10]. In the OPGC hydrogels, a dual-network structure was formed by the Schiff base bonds and hydrogen bonds, which endowed the hydrogels with ultrafast self-healing ability and gelation time.

**Figure 6:**
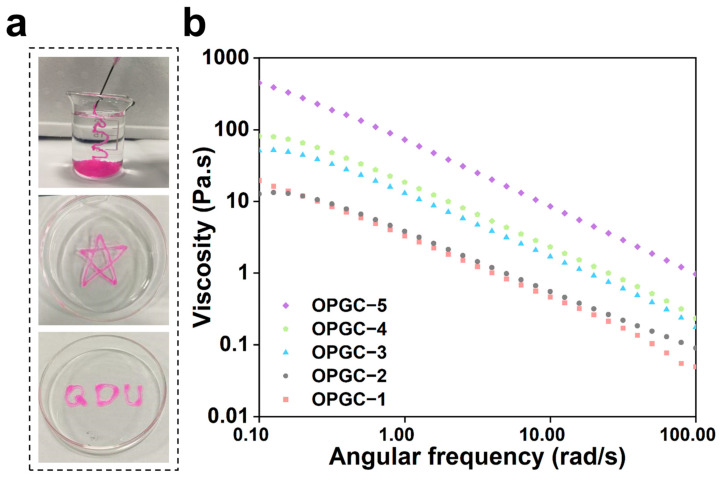
(**a**) Injectability of OPGC hydrogels; (**b**) Viscosity change of OPGC hydrogels.

Hydrogels for injection have received a lot of attention because they can be injected into any tiny site to deliver the desired gel shape [33]. Coarse peach gum has a hard texture and cannot be injected to form the desired shape. Excellent shear-thinning properties are exhibited by dynamic hydrogels based on Schiff base bonds and hydrogen bonds, which gives them excellent injectability. As shown in Figure 6a, extruded from the syringe, it was observed that the rhodamine B-stained OPGC hydrogels remained in a continuous gel state and could be obtained in any desired shape. The shear-thinning properties of hydrogels were further explored through rheological strain scanning tests.

As shown in Figure 6b, with a gradual increase in the shear rate, the viscosities of OPGC hydrogels with different CMCS contents all showed a significant decrease, proving that the OPGC hydrogels possessed the property of shear thinning (*p* < 0.05). As demonstrated, the hydrogels were endowed with good injectability by the shear-thinning property, which highlighted the practical benefits for applications requiring injection. The strong shear force generated by syringe extrusion broke the hydrogen bonds and Schiff base bonds in the hydrogel network. This action reduced the viscosity of the hydrogels and enabled them to flow continuously in a specific direction. When the shear force disappeared, a new hydrogel network was quickly formed through the reconstruction of the Schiff base bonds and hydrogen bonds. This also accounts for the ultra-fast 30 s self-healing capability of the OPGC hydrogels. Because of this property, the OPGC hydrogels are endowed with the potential for subcutaneous injection, which allows them to effectively deliver their protective and moisturizing effects.

### 2.6. Physiological Functional Activity of OPGC Hydrogels

**Figure 7:**
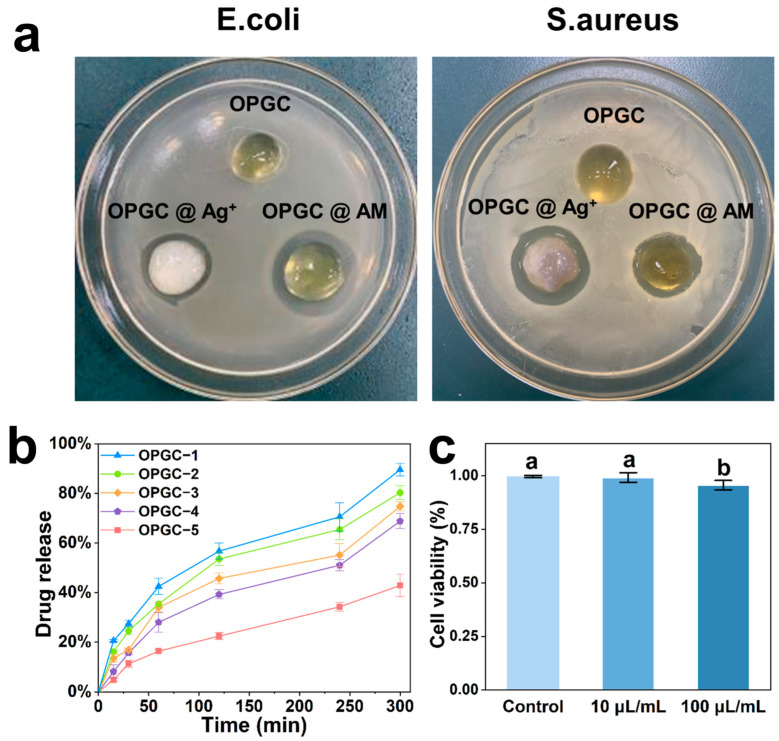
(**a**) OPGC hydrogels, OPGC @ Ag^+^ hydrogels, and OPGC @ AM hydrogels co-cultured with *E. coli* and *S. aureus* to form inhibition zones; (**b**) Release of amoxicillin from hydrogels. (**c**) Cell viability of L929 fibroblasts after 24 h of culture with medium-added OPGC hydrogels. Different letters indicate significant differences between the means (*p* < 0.05).

Hydrogels can be loaded with antimicrobial substances to obtain antimicrobial properties [34]. Although crude peach gum has antimicrobial capacity, its hard texture makes it less useful in practical applications. In contrast, the gel material constituted by OPGP achieves comparable antimicrobial ability by loading antimicrobial substances, but the gel hardness can be reduced, which makes it convenient for application. Amoxicillin (AM) is a common antibiotic in clinical practice, and silver ions (Ag^+^) are widely recognized for their antimicrobial properties; both are effective agents in combating infections [35]. Ag^+^ or AM can be encapsulated in situ by OPGC hydrogels. The antimicrobial properties of the resulting OPGC @ Ag^+^ hydrogels and OPGC @ AM hydrogels were studied using *E. coli* and *S. aureus*, which are commonly found in wounds and served as the experimental subjects. As shown in Figure 7a, the antibacterial zones tests demonstrated that *E. coli* and *S. aureus* can be effectively inhibited by the drug loaded in the drug-carrying hydrogels, which spread well. Conspicuous antibacterial zones were formed by the OPGC @ Ag^+^ hydrogels and the OPGC @ AM hydrogels. The antimicrobial effects of the drug-loaded OPGC hydrogels were noticeable, indicating that hydrogels can serve as excellent carriers for localized drug release.

Considering the high cost and narrow range of Ag^+^ use and the better bacteriostatic effect of OPGC @ AM hydrogels, OPGC @ AM hydrogels were chosen to test drug release from OPGC hydrogels. The drug release behavior of the hydrogels was explored through the monitoring of amoxicillin release by UV absorption. Hydrogels with different CMCS additions of OPGC hydrogels were loaded with 2 mg/mL amoxicillin, after which the absorbance value of the solution at 276 nm was measured. The release profile of amoxicillin is depicted in Figure 7b. As the CMCS addition increased, the OPGC hydrogels were able to load the drug better, thereby delaying its release. This was due to the formation of more Schiff base bonds, which resulted in a denser hydrogel network that allowed for the slow release of the drug. Therefore, the drug release rate from OPGC hydrogels can be controlled by adjusting the addition of CMCS. The OPGC hydrogels were not only endowed with excellent adjustable mechanical properties, self-healing ability, and injectability, but were also capable of being loaded with the desired drugs and successfully releasing them.

Biocompatibility is essential for hydrogel materials to be used in biological applications; hence, the cytocompatibility of OPGC hydrogels was explored in vitro. The OPGC hydrogels’ cytotoxicity was assessed using an MTT assay with L929 cells serving as the model cells. As shown in Figure 7c, after 24 h of co-incubation with cells, the survival rate of the L929 cells was found to exceed 99%, with no statistical difference compared to the control groups. This indicates the non-cytotoxicity of the hydrogels. Even when the sample concentration was increased tenfold, cell viability remained above 95%. Due to the utilization of natural polysaccharides as raw materials, no toxic effect on mouse cells was exerted by the OPGC hydrogels. Thus, the OPGC hydrogels exhibited good biocompatibility. This means OPGC hydrogels are suitable for applications such as cell repair with high biosafety requirements.

### 2.7. In Vivo Wound-Healing Evaluation

**Figure 8:**
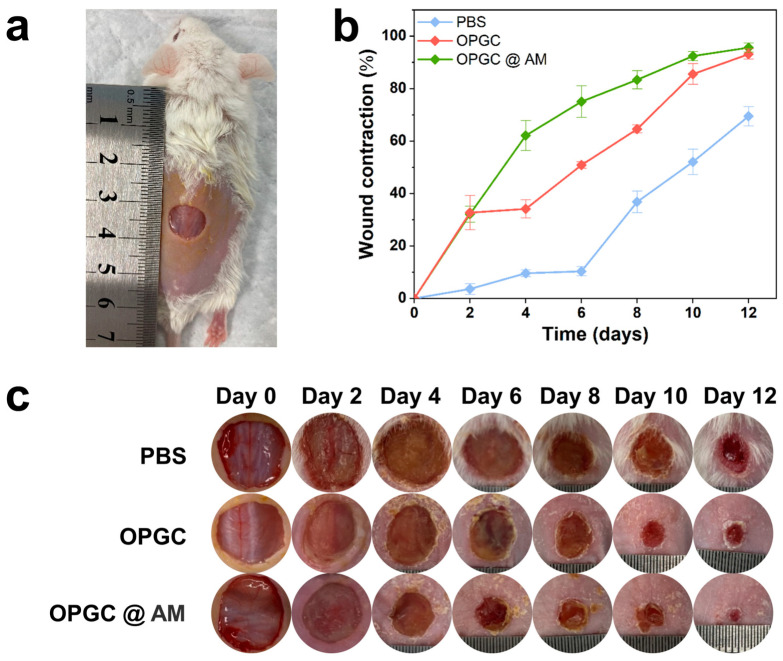
In vivo antimicrobial and wound-healing effects of hydrogels. (**a**) Photographs of skin defects in mice at day 0. Scale bar: Round diameter = 12 mm. (**b**) Wound shrinkage rate of mice at different treatment times. (**c**) Photographs of wounds treated with PBS, OPGC hydrogels, and OPGC@ AM hydrogels dressings.

*S. aureus* is one of the most common pathogens causing wound infections. To evaluate the potential of hydrogels as dressings in wound healing, a mouse dorsal total skin defect model was constructed and infected with *S. aureus* (Figure 8a). Then, the interventional effects of PBS, OPGC hydrogels, and OPGC @ AM hydrogels on the healing of infected wounds were systematically assessed by monitoring the dynamics of the wound area (Figure 8c) and quantitative analysis of the healing rate (Figure 8b) for 12 days (0/2/4/6/8/10/12 days). All hydrogel groups demonstrated better wound healing than the PBS control group owing to their good biocompatibility, as reflected by an increased rate of wound contraction, reduced exudation, and accelerated erythematous regression.

On the second day of treatment, the wound in the PBS control group developed a large amount of white pus, suggesting a severe infection. The OPGC hydrogels group exhibited no purulent exudation and demonstrated effective wound contraction (about 30% enhancement compared to the PBS control group). On the 12th day of treatment, the wound-healing rate in the OPGC hydrogels group was 92.7%. The wound-healing rate in the PBS control group was only 69.5% during the same period, with irregular wounds and blood oozing out. The results show that the OPGC hydrogels demonstrated an ability to promote wound healing. This was attributed to the moist environment provided by the hydrogel.

The OPGC @ AM hydrogels group demonstrated a superior state of sustained wound healing because of the loading of the antimicrobial drug amoxicillin. On the sixth day of treatment, the healing rate of the OPGC @ AM hydrogels group had reached 75%, while the PBS control group was only 10.3% during the same period. The slow-release behavior of amoxicillin in the hydrogels continuously maintains an effective antimicrobial concentration on the wound surface, thus promoting wound healing. The healing rate of the OPGC @ AM hydrogels group increased to 96% by the 12th day, which was higher than the OPGC hydrogels group and the PBS control group. The results show that the antibiotic-loaded OPGC hydrogel exhibited antimicrobial activity and accelerated wound healing. The results show that OPGC hydrogels could be used as good wound dressings.

### 2.8. Analysis of HE Staining in Wounded Skin

**Figure 9:**
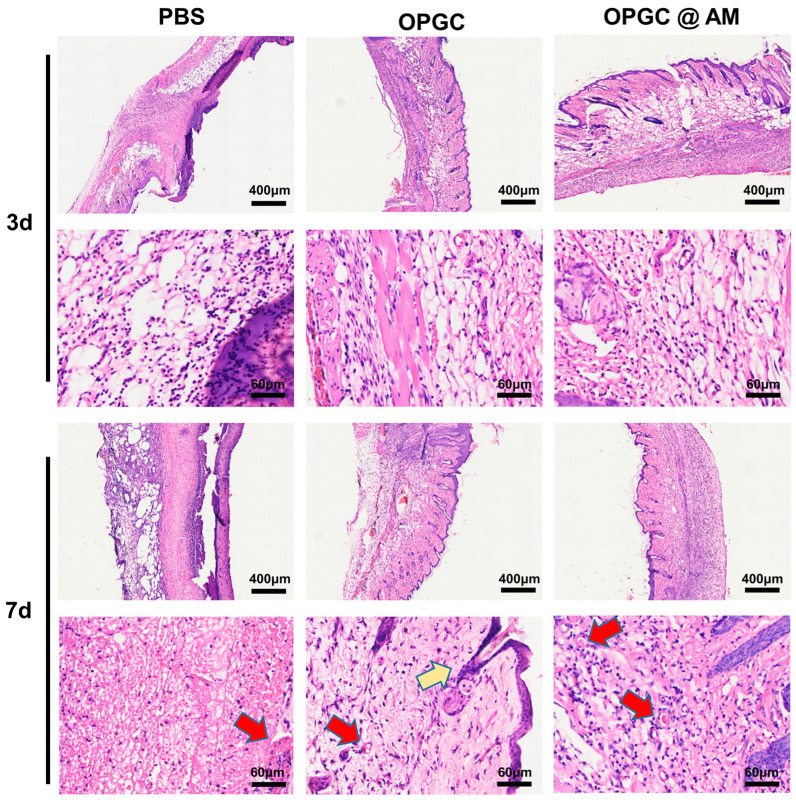
HE staining of mouse skin tissue sections. Neoplastic hair follicles were indicated by yellow arrows and neovascularization by red arrows.

Wound healing is a complex process involving multiple biological responses such as inflammatory modulation, neovascularization, and collagen deposition [36]. The HE staining method could be used to assess the integrity of the regenerated dermis and the epidermis and the status of neoplastic skin attachments. The HE staining analysis on the third day after treatment (Figure 9) showed that the new skin of the PBS control group had sparse connective tissue. Meanwhile, large numbers of inflammatory cells were observed to gather and distribute among the tissues, and the epithelium formed a distinct thick scar tissue. The skin tissue of the OPGC hydrogels group was relatively tight, and a small number of inflammatory factors were observed without an aggregated state, confirming the importance of a moist environment for epithelial regeneration. The stained images of the OPGC @ AM hydrogels group displayed neater skin, with further reductions in inflammatory factors, and connective tissue in an ideally tight state.

On the seventh day, the PBS control group showed some healing tendency, but the skin tissue was still loose and a large accumulation of inflammatory cells was visible. Comparatively, the OPGC hydrogels group had a more continuous structure at the dermal–epidermal junction, with a reduced infiltration of inflammatory cells and the appearance of early follicular germinal structures (yellow arrows). Meanwhile, a complete complex squamous epithelium had formed in this group, accompanied by the formation of capillary networks (red arrows). This phenomenon may be attributed to the maintenance of a moderately moist environment by the hydrogel, which promoted keratinocyte migration and fibroblast activation by regulating wound oxygen tension and osmotic pressure [37]. The OPGC @ AM hydrogels group further optimized the quality of tissue repair. The regenerated skin tissues (epithelium and dermis) showed a basic structure with tight and homogeneous connective tissue by the seventh day, and the generation of new blood vessels was clearly observed. This indicates that the antibiotic-loaded hydrogels facilitated tissue remodeling in infected wounds due to their more excellent antimicrobial properties.

### 2.9. Masson Staining Analysis of Wounded Skin

**Figure 10:**
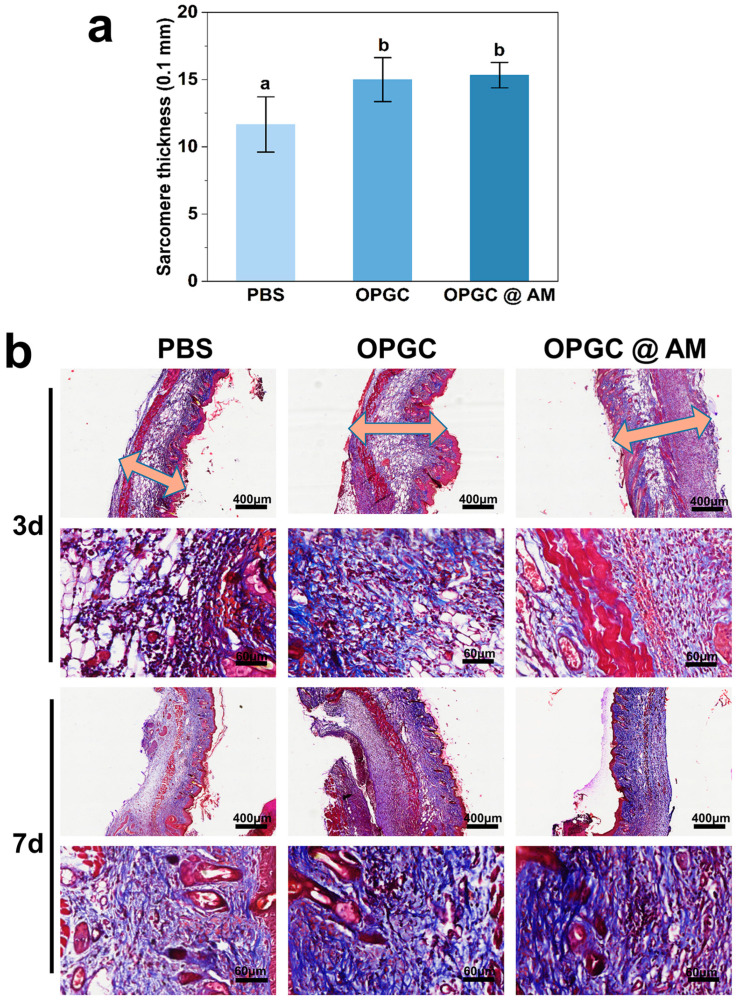
(**a**) Semi-quantitative analysis of sarcomere thickness. (**b**) Masson staining of mouse skin tissue sections. Different letters represent significant differences (*p* < 0.05).

The deposition and growth of collagen at the defective wounds were observed by Masson staining. Figure 10b shows that the PBS control group had a fragmented distribution of collagen fibers on the third day after treatment, whereas the OPGC and OPGC @ AM hydrogels groups demonstrated a distinctive tight arrangement of bright blue collagen fibers and a more orderly collagen deposition. Notably, the OPGC hydrogels group, although without loading drugs, showed collagen arrangement orderliness that was better than the PBS control group. Quantitative analysis results (Figure 10a) further revealed that the thickness of granulation in the OPGC @ AM hydrogels group and OPGC hydrogels group was significantly greater than that of the PBS control group (*p* < 0.05). This suggests that the hydrogel matrix and the drug synergized with the antimicrobial properties, thereby enhancing skin regeneration. The collagen distribution status on the seventh day showed that thick scarring was visible and collagen distribution remained sparse in the PBS control group. The OPGC hydrogels group had large amounts of tightly arranged bright blue collagen with no scar formation, while new hair follicle generation was observed. Granulation tissue in the OPGC @ AM hydrogels group was more densely deposited, and the collagen fiber bundles showed a highly ordered parallel arrangement pattern.

The above histological features were consistent with the results of HE staining, demonstrating that the use of OPGC hydrogels and OPGC @ AM hydrogels resulted in tight and orderly collagen distribution and excellent wound healing in the treatment of bacteria-infected wounds. The OPGC @ AM hydrogels exerted a synergistic antimicrobial effect through its matrix and the sustained release of amoxicillin, further promoting regenerative skin healing. Meanwhile, the moist environment provided by the dressing accelerated collagen synthesis, fibroblast proliferation, and rapid epithelialization, leading to inhibition of scar formation and promotion of wound recovery.

### 2.10. Immunofluorescence Staining Analysis of Wound Skin

**Figure 11:**
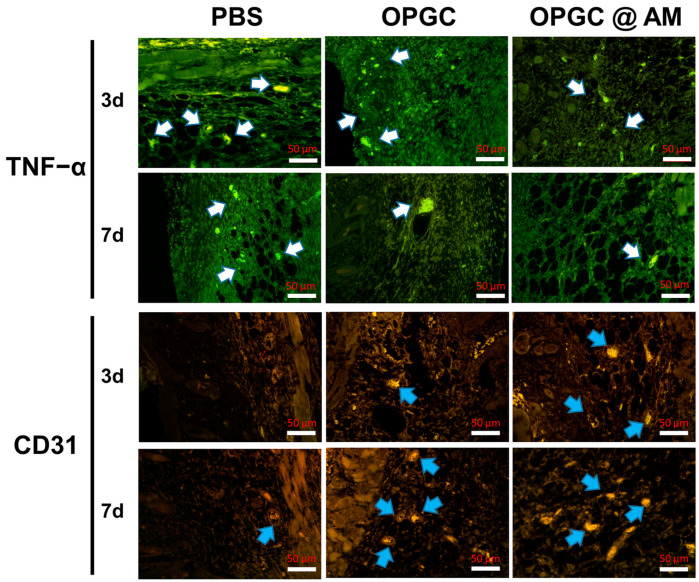
Photographs of skin wound tissues after immunofluorescence labeling with CD31 and tumor necrosis factor-alpha (TNF-α). Blood vessels are indicated by blue arrows and inflammatory factors are indicated by white arrows.

The efficacy of hydrogel dressings in preventing infections was tested using immunohistochemical analysis, selecting traumatic TNF-α as an indicator. As shown in Figure 11, TNF-α expression was higher in the PBS control group, indicating the occurrence of inflammation, whereas TNF-α expression was reduced in the OPGC hydrogels group and the OPGC @ AM hydrogels group, suggesting that the OPGC hydrogels can be effective in suppressing inflammation. CD31 is often used as an important indicator to assess angiogenesis. The micro-vessel density in the OPGC hydrogels group and OPGC @ AM group was obviously elevated compared to the PBS control group on the seventh day of the treatment course. The results indicate that the OPGC hydrogels and OPGC @ AM hydrogels prevented inflammation and accelerated angiogenesis by reducing the production of TNF-α and upregulating the expression of CD31, which effectively promoted the wound-healing process.

**Figure 12:**
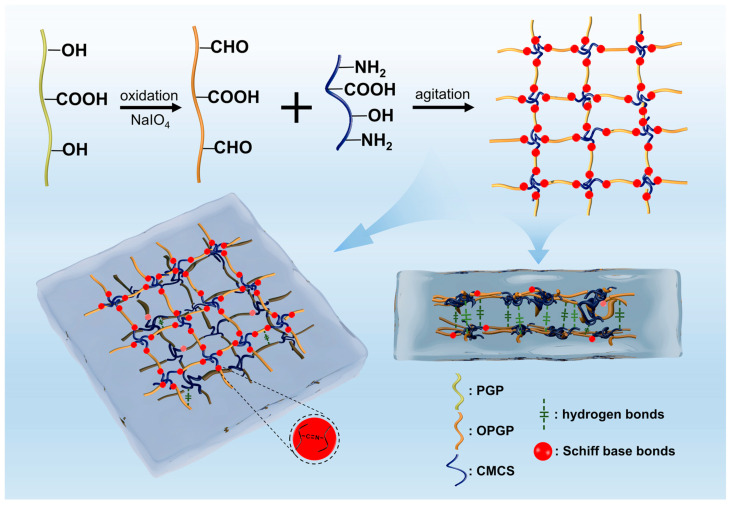
Schematic diagram of OPGC hydrogel formation mechanism.

Collectively, OPGC hydrogels exhibited excellent drug-carrying, self-healing, and wound-healing abilities in vitro and in vivo, and the gel-forming mechanism is shown in Figure 12. Natural PGP is rich in carboxyl and hydroxyl groups due to its macromolecular multibranch structure. The hydroxyl groups could be oxidized to aldehyde groups or form hydrogen bonds with other groups. CMCS was enriched with groups such as amino, carboxyl, and hydroxyl groups, such that amino groups and aldehyde groups could constitute Schiff base bonds. Schiff base bond formation requires no catalytic conditions and exhibited a fracture-reconnection function, which allows rapid reconnection after breakage to create new Schiff base bonds. This provided the hydrogels with self-healing and injectability properties. OPGP and CMCS self-assembled to create OPGC hydrogels with fast self-healing ability by utilizing aldehyde groups and amino groups constituting Schiff base bonds. Meanwhile, OPGP molecules formed a weak gel network through hydrogen bonding and hydrophobic interaction forces. The hydrogen bonds and Schiff base bonds together constituted the dual-network structure of the OPGC hydrogels, which enhanced the stability of the hydrogels.

## 3. Conclusions

In this work, injectable drug-carrying hydrogels with ultrafast self-healing ability were synthesized using PGP as the main raw material, then oxidatively modified and crosslinked with CMCS. The gel formation time, porosity, water loss, swelling, gel mechanical properties, and drug release rate of such hydrogels can be controlled by adjusting the amount of CMCS added, enabling the hydrogels to be tailored to fulfill a variety of needs in their application. Schiff base bonds were formed between the aldehyde groups of OPGP and the amino groups of CMCS, which, together with the hydrogen bonds among OPGP molecules, constructs the dense dual-network structure of the OPGC hydrogels. The OPGC hydrogels were endowed by this structure with excellent gel strength and ultra-fast self-healing ability as well as excellent injectability. In vitro and in vivo experiments demonstrated that OPGC hydrogels had excellent drug-carrying and slow-release ability. Meanwhile, it was found that OPGC hydrogels can inhibit wound infection and promote wound healing in mice. Hydrogels are virtually non-cytotoxic due to the use of natural materials. In conclusion, the use of OPGC hydrogels as a drug-carrying gel system with rapid self-healing ability has the potential to be applied for wound dressing in the medical field.

## 4. Materials and Methods

### 4.1. Materials

The PGP was purchased from Maya Reagent Co., Ltd. (Jiaxing, China). Sodium bicarbonate (NaHCO_3_), isopropyl alcohol, amoxicillin (AM), and silver nitrate (AgNO_3_) were acquired from Aladdin Biochemical Technology Co., Ltd. (Shanghai, China). CMCS (Mw = 369.47 K Da), HCI, NaOH, sodium periodate (NaIO_4_), agar, and NaCl were obtained from Macklin Biochemical Co., Ltd. (Shanghai, China). All reagents were of analytical grade and were utilized in accordance with their instructions.

### 4.2. Preparation of OPGP

In the extraction process of PGP, 15 g peach gum was dissolved in 500 mL deionized water, which was then stirred at 25 °C for 24 h. Afterward, 40 g NaOH (2 mol/L) was dissolved in the system, followed by alkalization at 60 °C for 2 h. After the reaction was completed, the solution was neutralized with 2 mol/L HCl and dialyzed in deionized water for 72 h to remove NaCl and other impurities. Finally, the desalination sample was concentrated using a rotary evaporator (Shanghai Quanjie Instrument Co., Ltd., Shanghai, China) and then freeze-dried as PGP powder.

To prepare OPGP, 5 g PGP powder was dissolved in 200 mL deionized water and stirred for 12 h. Then 3 mL of 0.5 mol/L NaIO_4_ was added to the PGP solution. The pH was equilibrated to 3.5 using HCl and NaHCO_3_ solution, then the solution was continuously stirred at 40 °C for 16 h. The reaction was carried out in the dark (avoid photo auto-oxidation). After 16 h, the reaction was terminated by adding 4 mL ethylene glycol. OPGP was precipitated by adding excess isopropanol, and the precipitate was collected by centrifugation. The collected precipitate was dried in a vacuum at 60 °C for 12 h to obtain the OPGP powder.

### 4.3. Determination of Aldehyde Groups Content in OPGP

To obtain a 0.25 mol/L hydroxylamine hydrochloride–methyl orange solution, 8.69 g hydroxylamine hydrochloride was dissolved in 75 mL deionized water and mixed with 3 mL of 0.05% methyl orange solution, adjusting the final volume to 500 mL. Then, OPGP powder was dissolved in the hydroxylamine hydrochloride–methyl orange solution (0.25 mol/L) and titrated with 0.1 mol/L NaOH solution. The titration was stopped when the color of the solution gradually changed from pink to yellow. Moreover, based on the titration curve, the volume of NaOH solution corresponding to the peak of the curve obtained was the volume of NaOH solution consumed at the end point of the titration, denoted as ∆V [38]. The determination of the aldehyde group content was repeated three times. The OPGP aldehyde group content was calculated as follows:

(1) $$[-\mathrm{CHO}]\;(\mathrm{mmol}/g)=\frac{\Delta V*0.001*n_{NaOH}}{W},$$

where ΔV (mL) is the calculated volume of NaOH solution consumed for the titration, n_NaOH_ is the molar concentration of NaOH solution, the W (g) is the mass of OPGP powder.

### 4.4. Synthesis of OPGC Hydrogels

To prepare the OPGC hydrogels, 300 mg OPGP powder was dissolved in 2 mL deionized water, adding CMCS at different mass ratios of 0.5/1/1.5/2/2.5 wt%. After the addition of CMCS, the solution was stirred rapidly for 1 min and then stood for 5 min to obtain the OPGC hydrogels. The OPGC hydrogels were named OPGC-1/OPGC-2/OPGC-3/OPGC-4/OPGC-5 according to the CMCS concentration (0.5/1/1.5/2/2.5 wt%).

In addition, OPGC hydrogels with in situ encapsulation of AgNO_3_ or AM were prepared. An amount of 300 mg OPGP was dissolved in 2 mL of 1 mg/mL AgNO_3_ solution or 2 mL of 1 mg/mL amoxicillin solution and stirred well. CMCS was added, the mixture was stirred rapidly to achieve homogeneity, and then stood to form hydrogels. OPGC @ Ag^+^ hydrogels and OPGC @ AM hydrogels were obtained.

### 4.5. Gelation Time of OPGC Hydrogels

The gelation time of the hydrogels was recorded using the flip-tube method. The hydrogel precursor was stirred well and immediately transferred to a centrifuge tube, and the tube was turned over continuously. The gelation time was noted when a gel sample was formed.

### 4.6. Fourier Transform Infrared (FTIR) of OPGC Hydrogels

The OPGP, PGP, CMCS, and OPGC hydrogels were subjected to analysis via a Nexus 470 Fourier transform infrared (FTIR) spectrometer (Thermo, Waltham, MA, USA). Separately, 2 mg samples were ground with 200 mg potassium bromide to achieve a thorough mixture, and the FTIR spectra of the samples were recorded in the range of 4000–500 cm^−1^, with resolution of 4 cm^−1^ and 16 scans.

### 4.7. Determination of Breaking Force and Gel Strength of OPGC Hydrogels

OPGC hydrogels with different additions of CMCS were prepared and then measured with a texture analyzer (CTA-10HD, Tianjin Chuangxing Co., Ltd., Tianjin, China). The test deformation was 1 mm with a 4.5 g trigger point. The probe was operated at a speed of 0.5 mm/s. Three consecutive tests were performed, the mean value was taken, and the standard deviation was calculated.

### 4.8. Rheological Analysis of OPGC Hydrogels

Rheological determinations were performed on a rheometer (MCR302, Anton Paar, Graz, Austria) by placing the hydrogels precursor on a 40 mm diameter plate and setting the temperature to 25 °C. The dynamic frequency range was 0.01~100 Hz at a strain of 1%. Three parallel samples were used for each group.

### 4.9. Physical Properties of OPGC Hydrogels

Water retention rate determination: The prepared OPGC hydrogels were weighed and recorded as m_0_ (g), and the hydrogels were placed in a 37 °C oven and weighed after 24 h and recorded as m_t_ (g). The formula for calculating the water retention rate was as follows:

(2) $$\mathrm{water}\;\mathrm{loss}\;\mathrm{rate}\;(g/g)=\frac{m_{0}-m_{t}}{m_{0}}\times 100\%,$$

Swelling rate determination: The prepared OPGC hydrogel samples were dried in an oven at 37 °C until constant weight, and then the dried samples were weighed and recorded as W_0_ (g). PBS buffer (0.01 mol/L, pH 7.4) was added to a 50 mL centrifuge tube and a hydrogel sample was placed in it, and the mixture kept at 25 °C for 12 h. The sample was removed and the liquid quickly wiped on the surface of the gel with filter paper; the weight was recorded as W_t_ (g). The formula for calculating the swelling rate was as follows:

(3) $$\mathrm{swelling}\;\mathrm{rate}\;(g/g)=\frac{W_{t}-W_{0}}{W_{0}}\times 100\%,$$

Water contact angle determination: OPGC hydrogels’ water contact behavior was examined using Theta Lite (Biolin Scientific, Gothenburg, Sweden).

### 4.10. Scanning Electron Microscopy Characterization of OPGC Hydrogels

The hydrogels were freeze-dried, and the micro-morphology of the hydrogels was observed using a scanning electron microscope (JSM-7800F, Nippon electronics, Hiratsuka, Japan) at an accelerating voltage of 2 kV. The hydrogels’ elemental distribution was then recorded by an EDS energy spectrometer (ESCALAB Xi +, Thermo Fisher, Czech Republic).

### 4.11. Measuring the Self-Healing Ability of OPGC Hydrogels

A piece of disc-shaped hydrogel was dyed with rhodamine B, and another piece was left undyed. After each piece was divided equally into four small pieces, two small pieces were taken from each piece of hydrogel to cross-combine and form a new disc shape. The four hydrogel pieces were observed until they formed a whole and could be picked up.

Microscopy was used to further observe the self-healing of the OPGC hydrogels. The hydrogels were placed on a slide after being cut to form gaps. The bright field was observed by IXplore epifluorescence upright microscope (OLYMPUS, Tokyo, Japan) (objective lens 10×), and the status of the hydrogels’ incision slits was photographed and documented.

### 4.12. Injectability Test of OPGC Hydrogels

A syringe was used to probe hydrogels’ injectability. Rhodamine B-stained OPGC hydrogels were loaded into a syringe, the syringe was squeezed to draw various shapes with the hydrogels, and the hydrogels were injected into the water. Moreover, the hydrogels were subjected to a shear thinning test: the viscosity change in the hydrogels was detected in the range of 1–100 s^−1^ at 25 °C.

### 4.13. In Vitro Antimicrobial Testing of Drug-Loaded OPGC Hydrogels

The antimicrobial properties of OPGC @ Ag^+^ hydrogels and OPGC @ AM hydrogels were verified by the circle of inhibition test using *E. coli* and *S. aureus* as bacterial models. Specifically, 100 μL bacterial suspension (10^8^ CFU/mL) was inoculated on the surface of an agar plate. Then 0.8 mL OPGC hydrogels, OPGC @ Ag^+^ hydrogels, and OPGC @ AM hydrogels were placed in full contact with the bacterial solution on the solid medium and incubated at 37 °C for 12 h. The diameter of the suppression ring was measured and photographed.

### 4.14. In Vitro Drug Release Assay of Drug-Loaded OPGC Hydrogels

A series of OPGC @ AM hydrogels were prepared with amoxicillin added at a concentration of 2 mg/mL, where the content of CMCS was 0.5/1/1.5/2/2.5 wt%. For each hydrogel 2 mL was taken in 20 mL PBS buffer (0.01 mol/L, PH 7.4) to explore amoxicillin release from OPGC @ AM hydrogels with different CMCS content. The absorbance value at 276 nm of the solution was measured every 15 min.

### 4.15. Cytotoxicity Tests

Mouse fibroblast L929 cells were cultured in DMEM containing fetal bovine serum double antibiotic (penicillin, streptomycin). Cultured cells were inoculated in 96-well plates with a final cell density of 8000 cells/well. The incubation was continued for 24 h. In a 96-well plate, 20 μL OPGC hydrogels extract was added to each well. After 24 h incubation, 0.5 μg/mL MTT was added and incubated for 4h. After the medium was removed, 200 μL DMSO was added to each well, and the incubation continue under dark conditions for 0.5 h. The absorbance value at 492 nm waws determined.

### 4.16. Construction of Infected Wounds of Full-Thickness Skin Defects in Mice

The mice were acclimatized for 5 days before the experiment. Ten mice were allocated to each of three groups, which were the PBS control group, the OPGC hydrogels group, and the OPGC @ AM hydrogels group. First, the mice were anesthetized and their back hair was shaved off. Each mouse’s dorsal region was sterilized, and a 10 mm diameter full skin wound was created on the dorsal skin of the mice. Subsequently, drops of 20 μL *S. aureus* suspension (2 × 10^8^ CFU/mL) were added to the wounds. Each group’s wounds were treated with 100 μL of PBS, OPGC hydrogels, and OPGC @ AM hydrogels.

### 4.17. Macro-Monitoring of Mouse Wounds and Calculation of Wound Closure Rates

The healing status of the wounds was photographed and recorded 2/4/6/8/10/12 days after treatment. The wound area was calculated using Image J. The trauma closure rate was calculated as follows:

(4) $$\mathrm{Wound}\;\mathrm{contraction}\;\mathrm{rates}=\frac{S0-Sn}{S0}\times 100\%,$$

where S_0_ represents the initial area of each wound (cm^2^) and S_n_ represents the wound area (cm^2^) on the nth day after administration.

### 4.18. Preparation and Staining of Mouse Skin Tissue Sections

On the 3rd and 7th day after treatment, full-thickness skin from the edge of the trauma was randomly collected from each group. Subsequently, the tissues were fixed, dehydrated, embedded, cut into 5 μm thick sections, and dried.

Tissue sections were dewaxed, rinsed in running water, and stained. HE staining: hematoxylin staining for 30 min, rinsed and stained with eosin for 5 min, and dehydrated. Masson staining: tissue sections were stained using a Masson staining kit, and the sections were immersed in potassium dichromate solution for 12 h. Sections were viewed with a microscope and stained images were captured and analyzed using a microimaging system.

For tumor necrosis factor-alpha (TNF-α) and CD-31 fluorescent staining, the tissue sections were first subjected to antigen repair. An autofluorescence quencher was added dropwise to the sections and then closed. After removing the closure solution, a drop of primary antibody was added to the section. After incubation, the secondary antibody of the corresponding primary antibody was added dropwise, and the sections were blocked. Finally, the sections were placed under a fluorescence microscope for observation and image acquisition.

### 4.19. Statistical Analysis

Statistical analysis of the data and significance testing was performed by ANOVA using SPSS 12.0 at a significant level of *p* < 0.05. The results were presented as mean ± standard deviation.

## Figures and Tables

**Table 1:** Aldehyde group content of oxidized peach gum polysaccharide (OPGP).

NaIO_4_: Peach Gum Polysaccharides (PGP)	[-CHO] Content (mmol/g)
0: 1	-
0.5: 1	0.78 ± 0.10 ^a^
1: 1	1.89 ± 0.13 ^b^
1.5: 1	2.13 ± 0.38 ^c^
2: 1	2.92 ± 0.14 ^d^
2.5: 1	3.30 ± 0.39 ^d^
3: 1	3.10 ± 0.15 ^d^

Different lowercase letters (a–d) indicate significant differences at different concentration. All *p*-values less than 0.05 were considered significant (*p* < 0.05).

## Data Availability

The original contributions presented in the study were included in the article. Further inquiries can be directed to the corresponding author.

## Supplementary Materials

The following supporting information can be downloaded at: https://www.mdpi.com/article/10.3390/gels11040274/s1, Figure S1: Gel formation at different concentrations OPGP.

## Abbreviations

The following abbreviations are used in this manuscript:

OPGC	Oxidized peach gum polysaccharide–carboxymethyl chitosan
OPGP	Oxidized peach gum polysaccharide
CMCS	Carboxymethyl chitosan
PGP	Peach gum polysaccharides
