# Supplementary material for: Preparation and Characterization of Dual-Network Multifunctional Hydrogels Based on Peach Gum Polysaccharides: Ultrafast Self-Healing Ability, Favorable Mechanical Tunability, and Controlled Release Properties
Source: Gels. 2025 Apr 6;11(4):274. doi: 10.3390/gels11040274 (PMC12026866; doi:10.3390/gels11040274)
Supplement: Supplementary file 1 [file gels-11-00274-s001.zip › gels-3546160-supplementary.pdf]

Supporting information

Figure S1: Gel formation at different concentrations of OPGP.

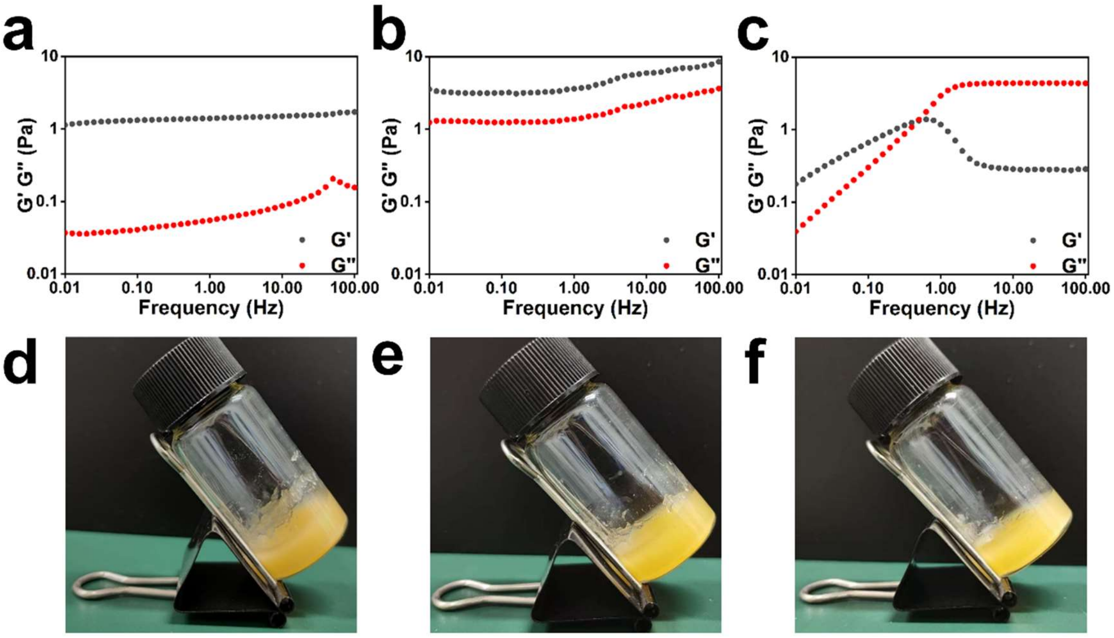

Figure S1:. (**a-c**) Storage modulus (G') and loss modulus (G'') of 150 mg/mL OPGP hydrogel, 200 mg/mL OPGP hydrogel, 250 mg/mL OPGP hydrogel. (**d-f**) 150 mg/mL OPGP hydrogel, 200 mg/mL OPGP hydrogel, 250 mg/mL OPGP hydrogel macro picture. The formation of OPGP hydrogel requires 3 hours.
